# Beta Lactamase Producing* Clostridium perfringens* Bacteremia in an Elderly Man with Acute Pancreatitis

**DOI:** 10.1155/2016/7078180

**Published:** 2016-01-24

**Authors:** Rashmi Mishra, Nupur Sinha, Richard Duncalf

**Affiliations:** ^1^Division of Pulmonary and Critical Care Medicine, Bronx Lebanon Hospital Center, 1650 Grand Concourse, Bronx, NY 10457, USA; ^2^Division of Pulmonary and Critical Care, Community Hospital of the Monterey Peninsula, 23625 Pacific Grove-Carmel Highway, Monterey, CA 93942, USA

## Abstract

*Clostridium perfringens* bacteremia is associated with adverse outcomes. Known risk factors include chronic kidney disease, malignancy, diabetes mellitus, and gastrointestinal disease. We present a 74-year-old man admitted with confusion, vomiting, and abdominal pain. Exam revealed tachycardia, hypotension, lethargy, distended abdomen, and cold extremities. He required intubation and aggressive resuscitation for septic shock. Laboratory data showed leukocytosis, metabolic acidosis, acute kidney injury, and elevated lipase. CT scan of abdomen revealed acute pancreatitis and small bowel ileus. He was started on vancomycin and piperacillin-tazobactam. Initial blood cultures were positive for* C. perfringens* on day five. Metronidazole and clindamycin were added to the regimen. Repeat CT (day 7) revealed pancreatic necrosis. The patient developed profound circulatory shock requiring multiple vasopressors, renal failure requiring dialysis, and bacteremia with vancomycin-resistant enterococci. Hemodynamic instability precluded surgical intervention and he succumbed to multiorgan failure. Interestingly, our isolate was beta lactamase producing. We review the epidemiology, risk factors, presentation, and management of* C. perfringens* bacteremia. This case indicates a need for high clinical suspicion for clostridial sepsis and that extended spectrum beta lactam antibiotic coverage may be inadequate and should be supplemented with use of clindamycin or metronidazole if culture is positive, until sensitivities are known.

## 1. Introduction

*Clostridium* species are Gram-positive, spore-forming, obligate anaerobic bacilli. Malignancies, renal insufficiency, and other chronic illnesses have been associated with* Clostridium perfringens* (*C. perfringens*) bacteremia [1]. This entity is associated with adverse outcomes, especially if not clinically suspected early in the course of disease [1]. We present a rare case of resistant* C. perfringens* bacteremia associated with acute pancreatitis. The purpose of this case report is to alert physicians to suspect* C. perfringens* bacteremia in elderly patients presenting with abdominal symptoms and sepsis. Additionally, providers should be aware that although rare,* C. perfringens* can produce beta lactamase which can complicate antibiotic management.

## 2. Case Presentation

A 74-year-old man was brought to our emergency room with altered mental status, abdominal pain, and multiple episodes of vomiting and diarrhea for one day. Review of systems was negative for any other systemic complaints including fever. His medical history was significant for hypertension. He had no prior history of diabetes or surgery and had no pertinent family history. Personal history included prior alcohol use for fifty years with a reported intake of approximately 3-4 beers daily.

On physical exam, he was tachycardic, hypotensive, and obtunded with cold extremities. His abdomen was tense and distended with sluggish bowel sounds. He was emergently intubated and aggressive resuscitation initiated for presumptive septic shock.

**Figure 1:**
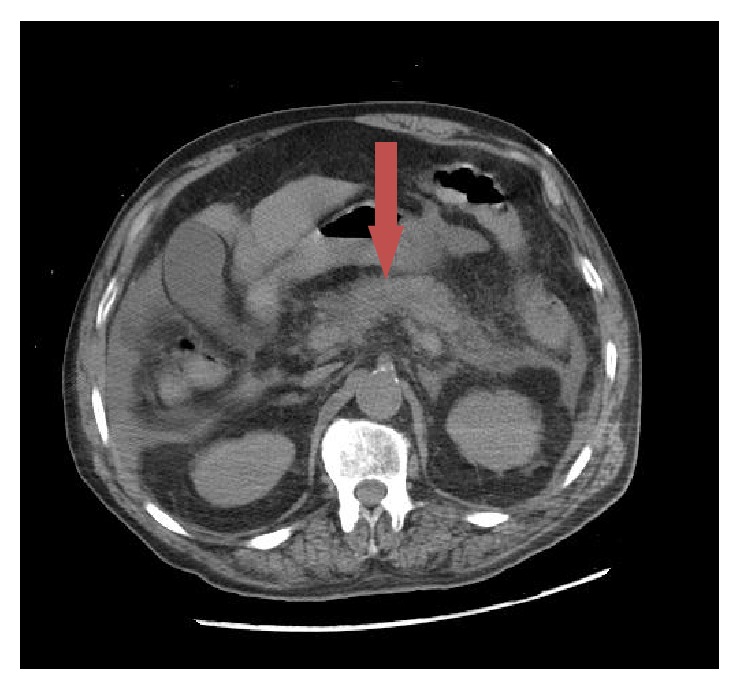
Axial cut of abdomen on day 1: arrow showing mildly edematous pancreas, with moderate peripancreatic infiltrative changes secondary to moderate acute pancreatitis.

Laboratory parameters revealed leukocytosis (12 × 10^3^/*μ*L), anion gap metabolic acidosis (pH 7.10 with anion gap of 31), acute kidney injury (creatinine level 2.6 mg/dL), elevated lipase (1088 U/L), and mildly elevated transaminases (aspartate aminotransferase level 95 IU/L, alanine aminotransferase level 256 IU/L, and alkaline phosphatase level 195 IU/L). Mean corpuscular volume (MCV) was 98 fL and there was no evidence of hemolysis. Of note, his HbA1c was 8.4% suggesting undiagnosed diabetes mellitus. Urine toxicology screen was negative and serum ethanol level was less than 10 mg/dL. Computerized tomography (CT) scan of the abdomen (Figure 1) revealed moderate peripancreatic infiltrative changes and dilated loops of small bowel consistent with moderate acute pancreatitis and small bowel ileus.

A blood culture drawn on admission was reported to be positive for* C. perfringens* on day five. Metronidazole and clindamycin were then added to the existing regimen of vancomycin and piperacillin-tazobactam. Subsequent antibiotic sensitivity testing of the* C. perfringens* isolate revealed beta lactamase positivity; hence clindamycin was continued to treat the* C. perfringens* bacteremia. Metronidazole was also continued for suspected colitis. Stool studies including* Clostridium difficile* toxin and cultures were negative. The patient continued to require vasopressor support despite appropriate antibiotics and continued aggressive medical therapy, with worsening renal failure requiring hemodialysis.

**Figure 2:**
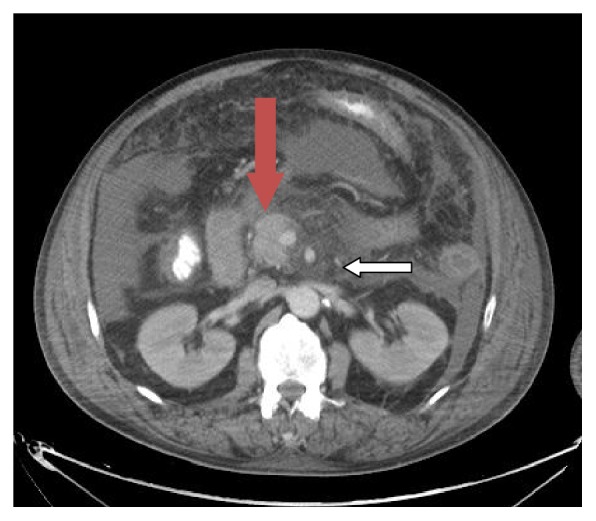
Axial cut of abdomen on day 7: red arrow pointing at a partially loculated fluid within the body of the pancreas. The white arrow shows an area of focal necrosis within the body of the pancreas.

A contrast enhanced CT of the abdomen on day seven revealed extensive acute pancreatitis with a new focus of likely necrotic pancreatitis in the pancreatic body with an associated 8.2 cm fluid collection consistent with a developing pseudocyst (Figure 2). Bilateral pleural effusions were also demonstrated. The patient's tenuous hemodynamic status precluded surgery. CT guided percutaneous aspiration of the pseudocyst fluid as well as pleural fluid was performed by interventional radiology. Cultures from these samples were subsequently negative. The remainder of the patient's hospital course was complicated by vancomycin-resistant enterococcus bacteremia and continued septic shock with worsening multiorgan failure and death.

## 3. Discussion

Advanced age increases the risk of clostridial infection independent of comorbidities which could be explained by age-related increase of clostridial species in the normal intestinal flora [2].* C. perfringens* is frequently isolated from the biliary tree and gastrointestinal tract [2–4].* C. perfringens* bacteremia has been reported after colonoscopy and gynecologic procedures [5, 6], and in association with choledocholithiasis in the absence of gallbladder stones and with normal common bile duct diameter [7].

Five subtypes of* C. perfringens* (A to E) exist and can produce as many as 12 different toxins. The 4 principal toxins of* C. perfringens* are alpha, beta, epsilon, and iota [8–10]. Alpha toxin can cause gas gangrene [11] as well as hemolysis and platelet destruction [12–14].* C. perfringens* bacteremia has been associated with intravascular hemolysis and death [9, 10, 15, 16]. Low MCV and hemolysed samples in a patient with fever should alert the clinician to the possibility of clostridial infection. Beta toxin is associated with necrotic enteritis [17]. Epsilon toxin is known to cause fatal enterotoxemia in sheep and other animals [18]. When injected intradermally, iota toxin causes an increase in capillary permeability and intradermal necrosis in guinea-pigs. Larger doses injected intravenously are lethal in animals [18].

*C. perfringens* bacteremia, especially with a penicillinase producing strain, is a rare clinical entity. Epidemiological studies examining* Clostridium* bacteremia have been conducted in Taiwan, Japan, Canada, and the United States.

A study from northern Taiwan [1] demonstrated an overall annual incidence of* C. perfringens* bacteremia of 0.97 per 100,000 population. Elderly patients with comorbid illnesses, especially renal insufficiency or malignancy, were at increased risk. The 30-day and attributed mortalities were 26.9% and 8.6%, respectively. Nosocomial acquired* C. perfringens* infection was a significant predictor of 30-day mortality. Most* C. perfringens* blood isolates were susceptible to the antibiotics tested. Resistance was observed in only seven out of ninety-three isolates, primarily to penicillin and clindamycin.

A review of all blood cultures drawn in a Japanese tertiary center from 2001 to 2009 demonstrated only 18 patients with* C. perfringens* bacteremia. Overall 30-day mortality was 27%. Septic shock at initial presentation was significantly associated with mortality [19]. A population-based surveillance of clostridial bacteremia among all residents of the Calgary Health Region (population 1.2 million) during 2000–2006 revealed a prevalence of clostridial bacteremia at 1.8/100,000 per year. Older age and multiple comorbidities, most importantly malignancy and Crohn's disease, were risk factors for acquiring* Clostridium* bacteremia.* C. perfringens* was the most common species isolated [2].

Review of blood cultures drawn in a rural hospital in Wisconsin from 1990 to 1997 yielded* Clostridium* infection in 0.12% with* C. perfringens* again, being the most common isolate (21.7%) [20].

Several studies have identified other conditions associated with the pathogenicity of* Clostridium* species and have demonstrated that failure to institute early, appropriate antimicrobial therapy may be associated with a poor outcome [2, 21, 22].

In another Taiwanese study, a review of 73 patients with clostridial bacteremia in an 11-year period identified diabetes mellitus and liver cirrhosis as the most common underlying comorbidities. Etiological species identified were* C. perfringens* (77%),* Clostridium bifermentans* (9%), and* Clostridium septicum* (4%).* Clostridium* bacteremia in patients with underlying liver cirrhosis and septic shock on initial presentation were poor prognostic factors [21].

The significance of positive blood culture for* Clostridium* was also studied in Israel. They found that growth of* Clostridium* species in blood cultures, even in the absence of one of the histotoxic syndromes, is often of clinical significance. Patients with* Clostridium* bacteremia were older, had a higher frequency of gastrointestinal disease, especially colorectal tumors, were more frequently associated with polymicrobial bacteremia, and had a higher mortality rate [22].

Our patient had several known risk factors for clostridial infection and subsequent mortality. He was older, apparently diabetic, had gastrointestinal disease, and presented in shock. However, none of the histotoxic syndromes associated with* Clostridium* infection were readily apparent. Although* C. perfringens* bacteremia was not initially suspected in our patient, he was treated with appropriate antibiotics.

Our isolate was reported to be beta lactamase producing. Based on the susceptibility report (see Table 1), our patient appeared to have a penicillinase producing organism as opposed to being a cephalosporinase producer.

The patient was initiated on piperacillin-tazobactam and vancomycin, both of which could be expected to have efficacy towards our isolate. Nevertheless, our patient deteriorated, requiring the addition of metronidazole and clindamycin. It is conceivable that penicillinase* C. perfringens* in vitro may predict cephalosporinase activity in vivo. Beta lactam resistance has been well studied in many pathogenic bacteria. In vitro susceptibility does not necessarily produce in vivo activity of an apparently appropriate antibiotic [23]. Unfortunately, we could not find any specific studies of this phenomenon related to* C. perfringens*. Early studies have demonstrated in vitro susceptibility of* C. perfringens* strains to vancomycin [24]. However a more recent study has shown that vancomycin is not bactericidal against* C. perfringens* [25].

## 4. Conclusion

Although rare, given the significant mortality of* C. perfringens* bacteremia, clinicians should be aware of the risk factors and presentation associated with this pathogen. We recommend immediate initiation of additional antibiotic coverage, for example, clindamycin or metronidazole, as soon as* C. perfringens* is isolated in culture, pending sensitivity. Data regarding clinical outcomes in beta lactamase producing Clostridia are scarce and may warrant further subgroup analysis. Furthermore, more microbiologic studies are required exploring in vitro and in vivo susceptibility patterns.

## Figures and Tables

**Table 1:** 

Antibiotic	Interpretation	MIC mcg/mL
Ampicillin/sulbactam	S	<1
Cefotaxime	S	4
Cefoxitin	S	<2
Ceftizoxime	S	<2
Chloramphenicol	S	4
Clindamycin	S	<0.5
Metronidazole	S	<0.5
Penicillin	R	
Piperacillin	S	<4
Tetracycline	S	<0.5

## Abbreviations

*C. perfringens*: * Clostridium perfringens*
CT: Computerized tomography
MCV: Mean corpuscular volume.
